# Repair of a Post-Hepatectomy Posterior Sectoral Duct Injury Secondary to Anomalous Bile Duct Anatomy Using a Novel Combined Surgical-Interventional Radiologic Approach

**DOI:** 10.1155/2013/202315

**Published:** 2013-09-12

**Authors:** Beth-Ann Shanker, Oliver S. Eng, Vyacheslav Gendel, John Nosher, Darren R. Carpizo

**Affiliations:** ^1^Department of Surgery, Rutgers-Robert Wood Johnson Medical School, New Brunswick, NJ 08903, USA; ^2^Division of Interventional Radiology, Department of Radiology, Rutgers-Robert Wood Johnson Medical School, New Brunswick, NJ 08903, USA; ^3^Division of Surgical Oncology, Department of Surgery, Rutgers Cancer Institute of New Jersey, Rutgers-Robert Wood Johnson Medical School, New Brunswick, NJ 08903, USA

## Abstract

A 64-year-old woman with a completely transected posterior sectoral duct following extended hepatectomy underwent a combined operative procedure with interventional radiology and surgery to restore biliary-enteric drainage. The anterior and posterior sectoral ducts were identified, and catheters were inserted into both systems. The posterior sectoral catheter was placed intraoperatively through a preoperatively placed sheath, and a new tunnel was created through the regenerated liver surface. Biliary-enteric anastomoses were created over the stents.

## 1. Introduction

Bile leakage following hepatectomy is a common and sometimes challenging clinical problem with incidences ranging from 3% to 15% [1–4]. Biliary leaks (or fistulas as sometimes called) predispose the patient to significant morbidity, which includes infectious complications due to bacterial contamination of the collecting bile, nutritional depletion, and electrolyte derangement in cases of high-volume leaks (>200 mL/day) secondary to the loss of enterohepatic circulation of bile. Extended left hepatectomy, central bisegmentectomy, and resection of the caudate lobe have a higher incidence of bile leakage as a result of damaging bile ducts from the caudate lobe and anomalous bile duct anatomy [5, 6].

Biliary leaks due to anomalous bile duct anatomy are some of the most challenging to manage, as they are often categorized as total or “complete” fistulae, which means they have no communication with the remaining biliary-enteric system. These fistulae will often not resolve without operative intervention. Once control of a complete fistula is obtained by placement of a percutaneous catheter to drain the relevant bile duct, cholangiography is necessary to define the area of liver that is involved. Surgical management choices are resection of the involved area of liver versus a biliary-enteric drainage procedure. In cases of major hepatectomy for malignancy, resection as a management option is often not feasible, as the patient cannot spare further loss of liver parenchyma; thus, biliary-enteric drainage is necessary. This operation poses significant technical challenges due to difficulties in localizing the site of the anomalous duct in the cut liver surface. 

Here we describe successful management of a patient with a complete biliary fistula involving the right posterior sectoral duct using a novel combined surgical and interventional radiologic approach. 

## 2. Case Report

**Figure 1:**
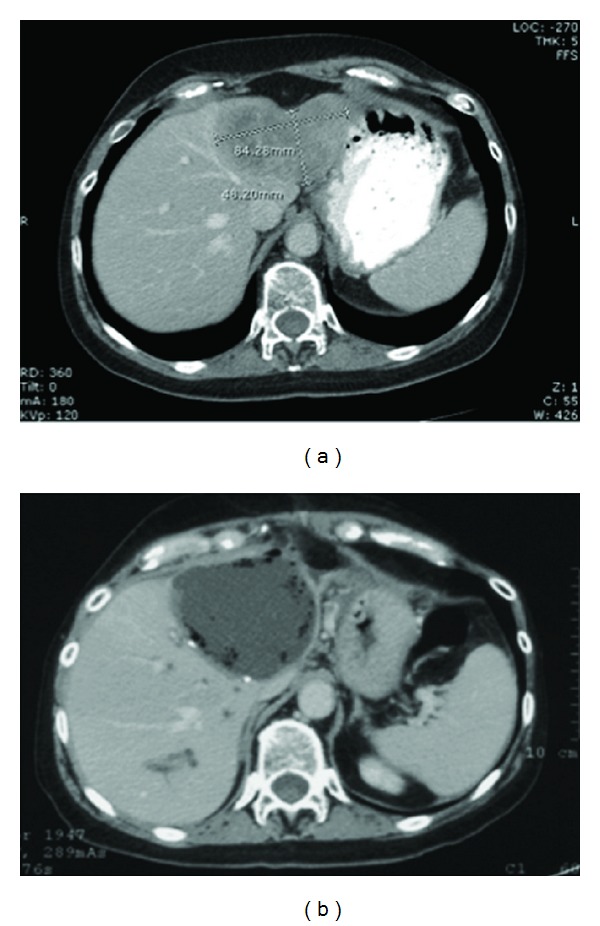
Preoperative and postoperative CT scan of patient. (a) Preoperative CT scan of this patient demonstrating a large metastatic breast cancer tumor (8.4 × 4.6 cm) located in the left hemiliver abutting the middle hepatic vein. An extended left hepatectomy including caudate lobectomy was performed with negative margins. (b) CT scan performed two weeks after hepatectomy demonstrating a large biloma located in the post-hepatectomy bed.

A 64-year-old woman was referred to our clinic with a 9 cm left liver mass, biopsy proven to be consistent with metastatic breast cancer, Figure 1(a). The patient had a 17-year history of metastatic invasive ductal carcinoma of the left breast to the small bowel and liver. Over a span of several years, she underwent multiple small bowel resections before developing a solitary left liver metastasis. Over time, it was observed that her tumor biology was unusual not only for its temporal nature (slow progression), but also its location (small bowel) for progression. Due to this unusual nature, as well as the fairly rapid growth of her liver tumor, resection was considered her best treatment option by a multidisciplinary group of oncologists. 

An extended left hepatectomy including caudate lobe resection and cholecystectomy was performed. The parenchymal transection in the area of segments 4b/5 went down to the bifurcation of the right and left pedicles in order to gain adequate tumor clearance. The left hepatic duct was divided separately with an endovascular stapler very close to the bifurcation of the right and left portal pedicles. During the operation, there were no immediate complications, including bile leak. The estimated blood loss was 150 mL, and the patient was discharged on the fourth postoperative day.

**Figure 2:**
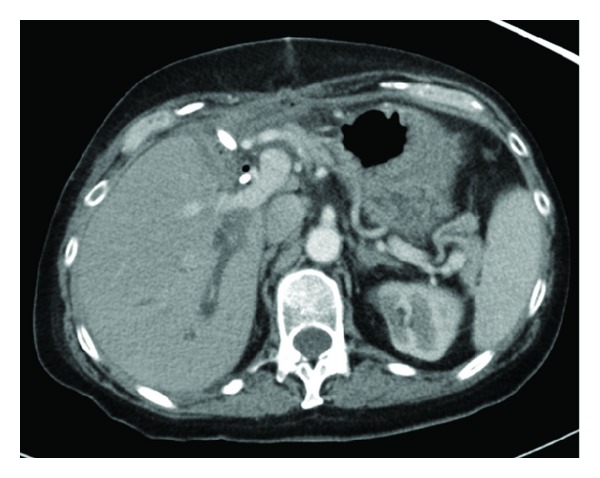
Isolated dilated right posterior sectoral duct. CT scan demonstrating dilated right posterior duct after adequate drainage of the biloma. Note the anterior ductal system is decompressed.

**Figure 3:**
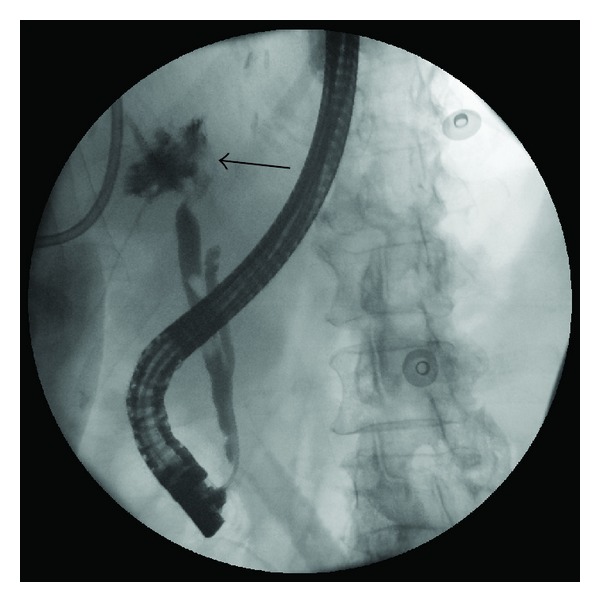
Post-hepatectomy ERCP. Post-hepatectomy ERCP demonstrating extravasation of contrast (black arrow) from the confluence of the right hepatic duct and left hepatic duct stumps.

**Figure 4:**
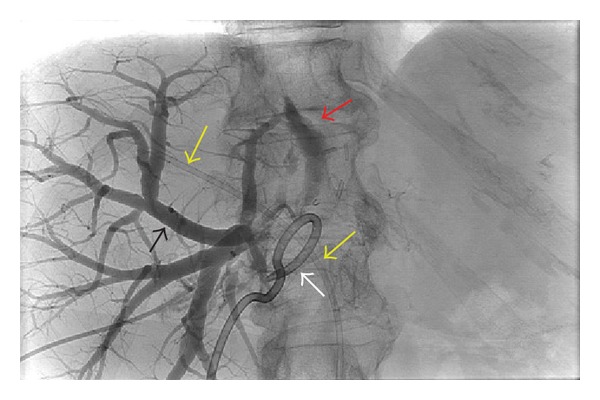
Confirmation of anomalous biliary anatomy. Transhepatic cholangiogram. Yellow arrows show endoscopic placed stent in right anterior sectoral duct. There is no contrast in the anterior sectoral duct or its branches. Black arrows show contrast in the posterior sectoral duct and filling of posterior duct and branches. White arrow shows extrahepatic pigtail placed catheter. Red arrow shows extravasation of bile. This cholangiogram demonstrates that the anterior and posterior ducts are not in continuity.

On postoperative day 15, she was admitted with abdominal pain, fevers, an elevated total bilirubin, and leukocytosis. CT scan demonstrated a collection in the hepatic fossa (Figure 1(b)), as well as a dilated right posterior bile duct (Figure 2). A percutaneous 10 Fr Felima pigtail drain was placed (Boston Scientific, Natick, MA) to drain the biloma. She then underwent endoscopic retrograde cholangiopancreatography (ERCP), where it appeared on cholangiogram that she had a leak from the left hepatic duct stump (Figure 3). A biliary endostent was inserted with the tip in the right anterior sectoral ductal system in an attempt to occlude the left hepatic duct stump. In followup, she was noted to continue to have a high amount of bilious output from the percutaneous drain, indicating an uncontrolled leak. Two weeks later, a transhepatic cholangiogram was performed through a catheter in the right posterior sectoral ductal system. This cholangiogram demonstrated that the right anterior sectoral duct containing the endoscopic stent was not in continuity with the posterior sectoral duct. The posterior duct was draining through the cut liver surface (Figure 4). We concluded there was anomalous biliary anatomy with the right posterior sectoral duct draining into the left hepatic duct. An external catheter was placed in this posterior duct. Over time, the percutaneous abdominal catheter stopped draining, indicating complete control of the fistula. The patient's sepsis was controlled and she recovered. The cut edge of the liver surface at the site of the transected posterior sectoral duct eventually sclerosed, making the catheter in the posterior sectoral duct no longer in communication with the abdominal cavity. A second operation to restore her biliary system and provide enteric drainage would be necessary. However, this operation posed a significant technical challenge to locate this aberrant duct in a reoperative field. It was decided that a combined interventional surgical approach would be necessary to identify the biliary anatomy intra-abdominally, create a new tract through the regenerated liver surface, and provide a stent to facilitate a new enteric anastomosis. 

### 2.1. Combined Surgical and Interventional Radiologic Approach

The tip of the catheter that was left in the right posterior sectoral duct was not placed in the extrahepatic space of the cut liver surface but rather was pulled into the liver, so we anticipated that this bile duct would have fibrosed in the several month period of time between operations. This would make it nearly impossible to find at reoperation. To facilitate identifying this catheter in the operating room, we first had the catheter injected with contrast in the interventional radiology department on the morning of surgery in an attempt to advance the catheter into the extrahepatic space. This no longer revealed an extravasation of contrast as when the catheter was initially placed, thus indicating there was no communication of the catheter with the peritoneal cavity. Next, the patient was moved to the operating room, where we performed an exploratory laparotomy; however, the sheath containing the posterior sectoral catheter was left in place to allow further manipulation in the operating room. At operation, we appreciated a large amount of fibrosis around the liver in the area of her previous biliary abscess. Next, the anterior biliary duct endostent was identified by palpation. Dissection around the anterior biliary duct led to the finding of a disruption of this duct at the confluence. This represented site of the leak of the left hepatic ductal stump was initially detected in Figure 4.

**Figure 5:**
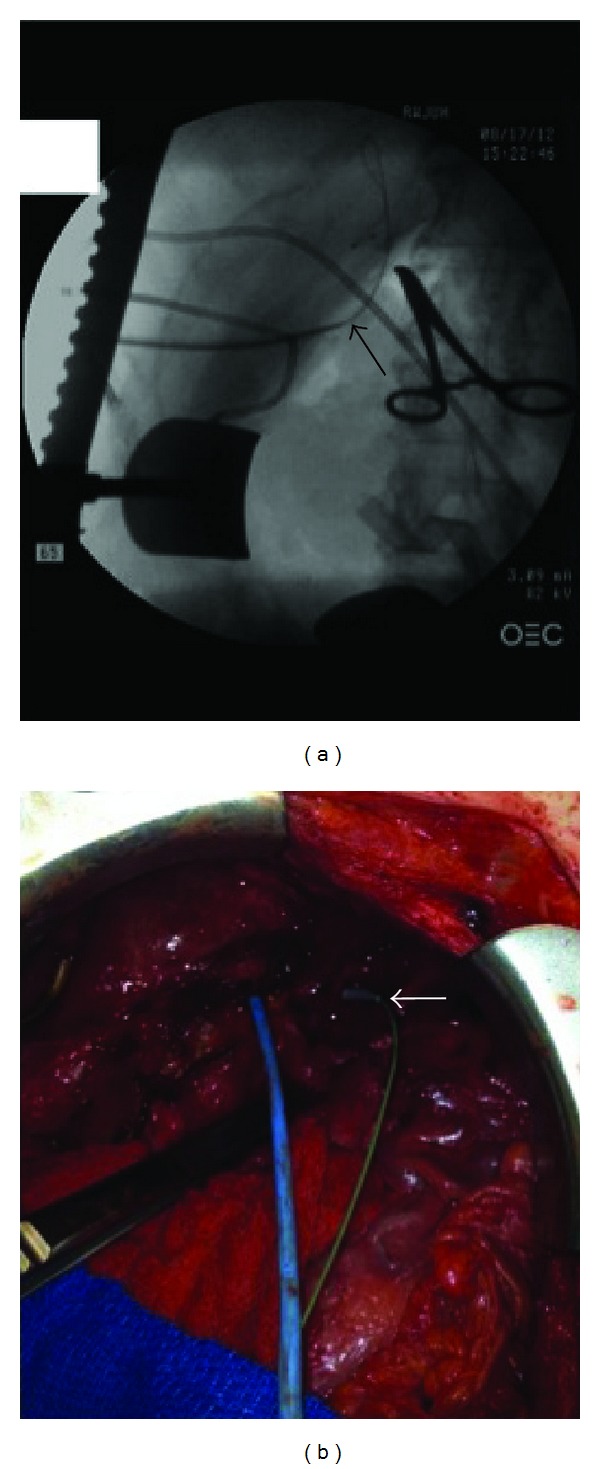
Intraoperative radiographically guided tunneling of biliary catheters. (a) Intraoperative radiograph showing interventional radiologist tunneling 16 gauge Colapinto needle through the previously placed posterior sectoral sheath (Cook Medical Inc, Bloomington, In). A guidewire traversing regenerated liver into the peritoneal cavity is demonstrated (arrow). (b) Operative field view with a catheter in the anterior ductal system inserted retrograde from the bile duct and out the liver surface and abdominal wall (arrow) and the posterior catheter inserted from an outside-in direction.

We next searched for the posterior sectoral catheter but could not identify or palpate it. This was expected. At this point, the interventional radiology team came into the operating room to provide fluoroscopic guidance for the location of the biliary catheter in the posterior duct. This revealed that the distance between the tip of the catheter and the cut liver surfaces was approximately 2-3 cm likely from regenerated liver. To traverse this distance, a tunnel would need to be made. Using the posterior sheath, we then placed a 16-gauge Colapinto needle with a 9 Fr Sheath (Cook Medical Inc., Bloomington, IN) and tunneled this out into the extrahepatic space, (Figure 5(a)). Using the same catheter system, we tunneled a catheter into the anterior ductal system retrograde from the duct orifice through the parenchyma and out the abdominal wall. We had two internal/external biliary catheters in both the anterior and posterior sectoral systems, (Figure 5(b)). We then fashioned a roux limb of jejunum and performed two separate anastomoses over these stents using interrupted sutures of 5-0 polydioxanone (PDS). The anterior anastomosis was a true hepaticojejunostomy with duct sewn to bowel; however, the posterior sectoral anastomosis was from the jejunum to a layer of fibrous tissue overlying the regenerated liver surface. As this was not a true hepaticojejunostomy, we buttressed this anastomosis using interrupted sutures of 3-0 PDS. The patient tolerated the procedure well and was discharged home on postoperative day 6. 

**Figure 6:**
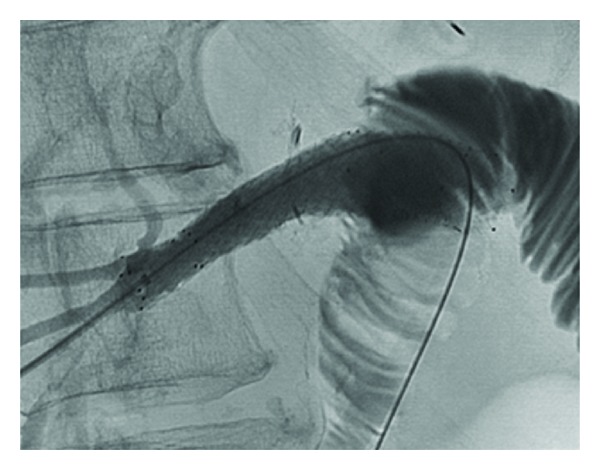
Biliary stenting of posterior sectoral anastomosis. Twelve weeks after operation to restore biliary-enteric drainage, interventional radiology placed overlapping SMART stents (Cordis, Miami Lakes, FL) across the posterior sectoral biliary-enteric anastomosis to prevent fibrosis of the tract not lined by bile duct epithelium.

In followup, the anterior internal-external biliary drain was removed after 4 weeks. The posterior internal-external drain was exchanged after 12 weeks for a permanent internal stent, which was composed of two overlapping SMART stents 14 mm × 6 cm and 14 mm × 4 cm (Cordis, Miami Lakes, FL, Figure 6) across the biliary enteric anastomosis. This was done to prevent future closing of the tract between the posterior sectoral duct and the jejunum that would likely happen, as there was approximately two centimeters of liver tissue not lined by biliary epithelium. 

## 3. Discussion

The association of major hepatectomy with increased bile leaks is well established in the literature [2, 6–8]. Left hepatectomy has been shown to be an independent risk factor for bile leaks [8]. Left hepatectomy and trisectionectomy with caudate lobe resection have challenging technical aspects including identification of the border between the caudate lobe and the right posterior section and the dividing line of the intrahepatic bile ducts [7]. Benzoni et al. examined their surgical complications in 134 patients with liver resections secondary to hepatocellular carcinoma (HCC) and 153 patients with liver resections secondary to metastasis. They found a significantly higher rate of bile leaks in patients after major hepatectomy, left hepatectomy, trisegmentectomy, and bisegmentectomy [2]. The majority of these bile leaks seal spontaneously, as these are considered “partial” leaks because they remain in communication with the remaining biliary-enteric system. In a retrospective review of 363 hepatectomies for cancer, Tanaka et al. reported an overall leak rate of 7.2% (26/363) with the majority (18/26, 69%) sealing within two weeks. Eight patients required some type of intervention, with two of the eight requiring reoperation. Neither required reresection or biliary bypass [6]. 

**Figure 7:**
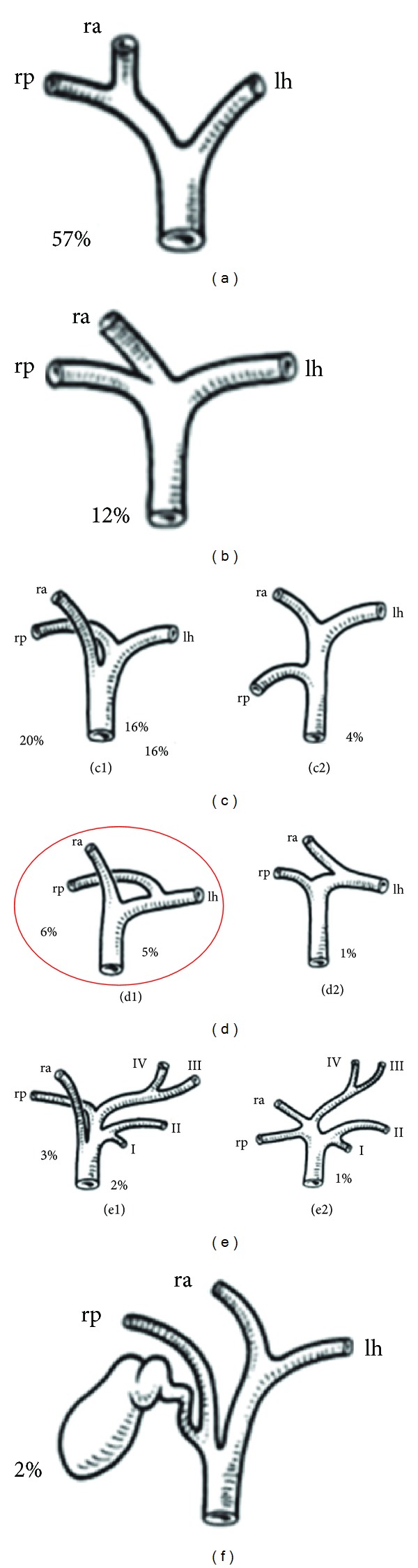
Normal hepatic duct anatomy and common variations (Couinaud 1957). (a) Typical anatomy. (b) Triple confluence. (c1) Right anterior draining into common hepatic duct. (c2) Right posterior duct drainage into common hepatic duct. (d) Right sectoral duct into the left hepatic ductal system. Red circle indicates the anatomy of the patient in this study. (e) Absence of confluence. (f) Absence of right hepatic duct. Drainage of right posterior duct into the cystic duct. The circled image corresponds to the biliary anatomy of this patient. Adapted and with permission to publish from Surgery of Liver, Biliary tract, and Pancreas, L. H. Blumgart editor. (2007, Saunders Elsevier: Philadelphia page 44).

Infrequently, a bile leak is considered a “complete” biliary leak/fistula, in which a segment or sector is completely separated from the remaining biliary-enteric system. These complete leaks/fistulas are often the result of aberrant hepatic ductal anatomy, most commonly when the right posterior sectoral duct drains via the left hepatic duct and the patient undergoes a left hepatectomy, as in this case. The classification of aberrant duct anatomy is well established (Figure 7), although the incidence of these types varies depending on studies of cadavers or imaging studies [9, 10]. The majority of the aberrant types involve the right system and its configuration with the common hepatic duct or left hepatic duct. The incidence of the type in this case in which the right posterior sectoral duct drains directly into the left hepatic duct before it joins the right anterior sectoral duct to form the common hepatic duct varies from 4–19% [5, 11, 12]. When a leak occurs after hepatectomy due to aberrant ductal anatomy such as this, it must be managed either by resection of the involved segment(s) or a biliary-enteric drainage procedure. Another option is to allow the involved liver to atrophy due to chronic cholestasis, but this can lead to septic complications of cholangitis. Biliary-enteric drainage is technically challenging, as one must locate the aberrant duct at the cut liver surface. Given the frequency of these variants, it is surprising that there is neither literature documenting the frequency of these types of leaks, nor the any description of their operative management.

Obviously, the best strategy for this problem is to avoid it altogether, which would require cholangiography being performed on all major hepatic resections, if not all left or extended left hepatic resections. Endoscopic or percutaneous cholangiography involves another procedure that carries its own set of potential complications. Only until recently have improvements in MR cholangiography made it possible to potentially anticipate this problem preoperatively. A growing body of research in the arena of living donor liver transplantation (LDLT) has studied biliary anatomy since donor safety is of particular concern. Approximately 250 cases of LDLT are performed yearly, with a range of 2.4% to 5.3% experiencing biliary complications. Preoperative evaluation had included magnetic resonance cholangiography (MRC) and computed tomography cholangiography (CTC). Conventional MRC may fail to delineate normal intrahepatic ducts because of a poor signal to noise ratio and limited spatial resolution. Wang et al. reviewed the recent literature on image evaluation of bile ducts. They found in a study of 111 LDLT donors that MRC accurately portrayed the anatomy of the biliary system in 88.3% of the subjects. CTC was found to be concordant with surgical findings in 23/24 LDLT patients for right liver donors. Overall, the studies on preoperative MRC and CTC are fairly limited, and in the realm of LDLT, surgeons typically rely on intraoperative cholangiography [13]. 

Taketomi et al. established an imaging and technical protocol in 2005 to define biliary anatomy and reduce the percentage of biliary leaks in LDLT. Despite preoperative CT cholangiography, they routinely obtained intraoperative cholangiograms after 2005, in addition to making other technical changes. They report a significant decrease in bile leaks since the introduction of their protocol [14]. However, these intraoperative cholangiograms are also limited by a two-dimensional representation of biliary anatomy [13]. 

The fact that the rate of biliary complications and leaks has not changed over the past decade indicates that preoperative imaging, intraoperative cholangiography, and the use of sealants are still limited in their ability to detect aberrant anatomy and prevent leaks. The management of biliary leaks is well studied in patients after laparoscopic and open cholecystectomy. In this setting, multidisciplinary approaches to manage such complications have been well described between the gastroenterologists and the surgeons. For cystic stump leaks, ERCP is successful as a tool for both diagnosis and therapeutic management with stent placement [4]. Yet, even in the cases of injury during cholecystectomy, aberrant anatomy of the right posterior duct has made it impossible to identify the leak via ERCP if the injured duct is not in communication with the main bile channels. These injuries require definitive management with a roux-en-Y hepaticojejunostomy [15]. Jarnagin and Blumgart reviewed operative repair of bile duct injuries involving the hepatic duct confluence [16]. Prior to any attempt at operative repair, they advocated for percutaneous transhepatic cholangiography to define the injury, angiography if there is concern for vascular injury, drainage of fluid, and biliary decompression if patients are septic. The fundamental principles cited for biliary reconstruction at the confluence include identification of healthy bile duct mucosa, roux-en-Y anastomosis 70 cm proximal to the enteroenterostomy, and a direct mucosa to mucosa anastomosis. In our particular patient, identifying healthy mucosa of the anterior and posterior sectoral ducts was challenging in the dense fibrotic and regenerate hepatic tissue. The combined procedure with interventional radiology and intraoperative fluoroscopy and placement of new biliary catheters allowed us to identify these ducts so that an adequate biliary-enteric anastomosis was performed.

In summary, we describe a novel combined approach in which interventional radiology combined with surgery leads to a successful repair of an aberrant right posterior sectoral duct following extended left hepatectomy. While interventional radiologists and hepatobiliary surgeons often work closely in hepatobiliary units, this is the first time that a surgical biliary bypass procedure has been described as a combined procedure with interventional radiology. Surprisingly there are no reports of techniques to overcome the problem of repairing a complete biliary fistula involving a transected duct at the edge of transection of the liver parenchyma after-hepatectomy. Due to the regeneration of liver tissue at the cut surface, it is impossible to surgically drain without the assistance of interventional radiology. 

It might be possible to anticipate this anomalous anatomy through preoperative MR Cholangiography. This raises another issue of what to do if an aberrant posterior sectoral duct is revealed by preoperative MR cholangiography. It might be very difficult to locate such a duct during parenchymal transection even when armed with such knowledge preoperatively. In such a situation, we would advocate a combined surgical and interventional approach as we have described, where the patients have a catheter placed into the posterior sectoral duct preoperatively and advanced into the left hepatic ductal system, such that this duct can easily be located during parenchymal transection and an anastomosis can be made with a roux limb of jejunum. At this time, we would advocate routine MR cholangiography for any extended left hepatic resection.
